# Spatial Distribution and Health Risk Assessment of Potentially Toxic Elements in Surface Soils of Bosten Lake Basin, Central Asia

**DOI:** 10.3390/ijerph16193741

**Published:** 2019-10-04

**Authors:** Long Ma, Jilili Abuduwaili, Wen Liu

**Affiliations:** 1 State Key Laboratory of Desert and Oasis Ecology, Xinjiang Institute of Ecology and Geography, Chinese Academy of Sciences, Urumqi 830011, China; Jilil@ms.xjb.ac.cn (J.A.); liuwen@ms.xjb.ac.cn (W.L.); 2 Research Center for Ecology and Environment of Central Asia, Chinese Academy of Sciences, Urumqi 830011, China; 3 University of Chinese Academy of Sciences, Beijing 10049, China

**Keywords:** arid land, classical linear model, geographically weighted regression, influencing factors, soil geochemistry

## Abstract

A geographically weighted regression and classical linear model were applied to quantitatively reveal the factors influencing the spatial distribution of potentially toxic elements of forty-eight surface soils from Bosten Lake basin in Central Asia. At the basin scale, the spatial distribution of the majority of potentially toxic elements, including: cobalt (Co), chromium (Cr), copper (Cu), nickel (Ni), lead (Pb), thallium (Tl), vanadium (V), and zinc (Zn), had been significantly influenced by the geochemical characteristics of the soil parent material. However, the arsenic (As), cadmium (Cd), antimony (Sb), and mercury (Hg) have been influenced by the total organic matter in soils. Compared with the results of the classical linear model, the geographically weighted regression can significantly increase the level of simulation at the basin spatial scale. The fitting coefficients of the predicted values and the actual measured values significantly increased from the classical linear model (Hg: *r*^2^ = 0.31; Sb: *r*^2^ = 0.64; Cd: *r*^2^ = 0.81; and As: *r*^2^ = 0.68) to the geographically weighted regression (Hg: *r*^2^ = 0.56; Sb: *r*^2^ = 0.74; Cd: *r*^2^ = 0.89; and As: *r*^2^ = 0.85). Based on the results of the geographically weighted regression, the average values of the total organic matter for As (28.7%), Cd (39.2%), Hg (46.5%), and Sb (26.6%) were higher than those for the other potentially toxic elements: Cr (0.1%), Co (4.0%), Ni (5.3%), V (0.7%), Cu (18.0%), Pb (7.8%), Tl (14.4%), and Zn (21.4%). There were no significant non-carcinogenic risks to human health, however, the results suggested that the spatial distribution of potentially toxic elements had significant differences.

## 1. Introduction

The influence of human activities on the surface of the Earth has continued to increase over the past hundred years [1,2,3]. Trace elements in surface agricultural soils are easily influenced by human activities via atmospheric deposition, irrigation, and fertilizer usage [4,5]. Long-term inputs of potentially toxic elements will lead to the enrichment of ecosystems and will be increasingly toxic to organisms [6,7,8]. It is not surprising that soil research has increased exponentially in recent decades [9,10]. However, it is undeniable that previous research areas have been mainly concentrated in developed regions [11,12,13].

From the aspect of the research methods used for pollution of potentially toxic elements, classic statistical methods have been used to reveal the possible influencing factors [14,15,16]. However, many researchers have given more consideration to influences on the distribution of potentially toxic elements from a quantitative point of view [17,18,19,20]. The geographical setting and climatic conditions of Central Asia led to ecological fragility and low carrying capacity in this region [21]. Research often focuses on the influence of human activities on land use and land cover changes [22,23], and soil degradation in Central Asia [24,25,26,27]. However, studies on potentially toxic elements in soils in this region have been scarce [28]. By studying the current risk state of potentially toxic elements in soils in this typical region, we can obtain a better understanding of the distribution of potentially toxic elements with different influential factors in Central Asia. The results will reveal the potentially toxic elements that are susceptible to human activities and will provide significant information for resource protection and management in the future.

The Bosten Lake region has begun to experience large-scale development. Due to the importance of the Bosten Lake region, studies on the paleoclimatic [29] and paleoenvironmental evolution [30] of the region and the pollution caused by polycyclic aromatic hydrocarbons [31,32], heavy metals [33,34], and organochlorine pesticides [35] have been performed for Bosten Lake sediments. In this study, using classic statistical methods and geographically weighted regression modeling [36,37,38,39], the influencing factors of potentially toxic elements in surface soils in this region, combined with a quantitative method and the assessment of the pollution of potentially toxic elements, are revealed in a typical arid area (Bosten Lake region) in Central Asia.

## 2. Materials and Methods

### 2.1. Regional Settings

**Figure 1:**
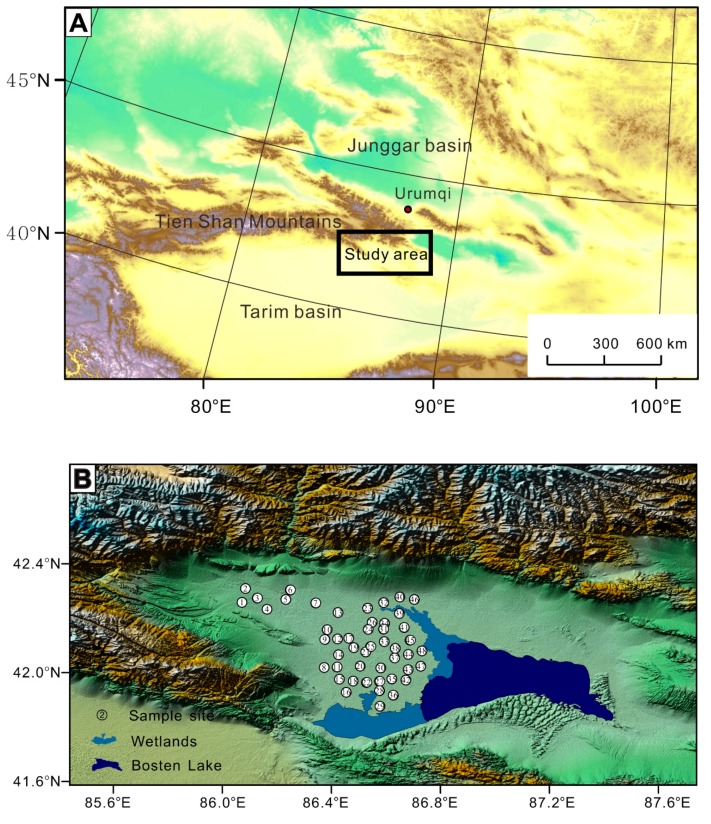
The geographical location of the Bosten Lake region (**A**) and the sampling sites (**B**).

The Bosten Lake basin lies between the Tian Shan Mountains and the Taklamakan Desert (Figure 1) and has a typical arid climate [40]. To the north is Aragou Mountain (Mt.) with a peak of 4000–4300 m above sea level. Hora Mt. and Kuruktag Mt. are to the south with elevations of 3000–2000 m. Erbin Mt. is to the west with an elevation greater than 4300 m. There are dry hills to the east at altitudes below 2000 m. In the 1960s, the farmland area was 1174.86 km^2^ and by the early 1990s, the area increased by 760.41 km^2^ [41]. The total annual precipitation is only 76.1 mm; however, evaporation amounts to 2000 mm year^−1^ [42]. There are four counties, including Yanqi, Hejing, Heshuo, and Bohu, in the Bosten Lake region. Over the past half-century, the economy has developed rapidly. We calculated the sums of several economic variables, including the year-end population, gross domestic product (GDP), total sown area for farm crops, and the number of industrial enterprises, to confirm the rapid growth in the region. For example, the GDP sharply increased from 5.6 × 10^6^ Chinese Yuan (CNY) in 1949 to 3.8 × 10^8^ CNY in 2004, which reflected the dramatic increase in human activities.

In addition, Bosten Lake is a basin that was previously the largest inland freshwater lake in China, with a water surface area greater than 1000 km^2^ [43]. Because various basin materials are not exported from the basin and are instead discharged into lakes, Bosten Lake has undergone significant changes under the pressure of human activities, for example, the salinity has increased from 0.38 to 1.87 g L^−1^ [44]. Changes in the geochemical composition of surface soils in the basin directly control the materials, ecosystem structure, and ecological security of Bosten Lake.

### 2.2. Sampling and Analyses

Surface soil samples (0–5 cm) were collected at 48 sampling sites in the Bosten Lake basin (Figure 1). At each sampling site, the soil sample was mixed with 5 sub-samples that were distributed at the center and four points of a 2 m × 2 m square with a sampling style of “×” form. The content of total organic matter (TOM) was confirmed via oxidation by using the potassium dichromate method [45]. Bulk soils with masses of ~0.125 g were ground through a 200-μm size mesh, digested with HF–HNO_3_–HClO_4_, and analysed using inductively coupled plasma atomic emission spectroscopy for the elements (Fe and V) and inductively coupled plasma mass spectrometry for the potentially toxic elements: As, Cd, Co, Cr, Cu, Hg, Ni, Pb, Sb, Tl, and Zn.

### 2.3. Data Analyzing

A principal component analysis potentially assisted in identifying the probable factors influencing the distribution patterns of pollution [46,47,48]. Pearson correlation analysis [49] was used to reveal the inter-relationships among the Fe and the potentially toxic elements (As, Cd, Co, Cr, Cu, Hg, Ni, Pb, Sb, Tl, and Zn). Kolmogorov–Smirnov (K–S) test was also applied to conduct normality tests.

A classical linear model assumes that the estimated coefficient for the independent variable is constant [50]. The model presumes that the value of Y has a linear correlation with a set of environmental variables (X_i_) as follows:

(1) $$y=\beta_{0}+\sum_{i=1}^{n}\beta_{i}x_{i}$$

In contrast, geographically weighted regression is a traditional method that extracts a set of local parameters [51,52] and shows a relationship that varies in space, which can be written as:

(2) $$y_{j}=\beta_{0}(u_{j},v_{j})+\sum_{i=1}^{p}\beta_{i}(u_{j},v_{j})x_{ij}$$

where u_j_, v_j_ represents the coordinates for each location j, β_0_(u_j_, v_j_) represents the intercept, and β_i_(u_j_, v_j_) is a local parameter for variable X_i_ at location j. Details on the geographically weighted regression can be found in the user manual for GWR4 software package version 4.09 [53]. To evaluate the modeling results, the parameters, including the Nash–Sutcliffe efficiency (NSE), percentage bias (PBIAS), and root mean square error (RSE), were calculated as follows [54]:

(3) $$NSE=1-\frac{\sum_{i=1}^{n}{\left(X_{i}-{\hat{X}}_{i}\right)}^{2}}{\sum_{i=1}^{n}{\left(X_{i}-\bar{X}\right)}^{2}}$$

(4) $$RSR=\frac{\sqrt{\sum_{i=1}^{n}{\left(X_{i}-{\hat{X}}_{i}\right)}^{2}}}{\sqrt{\sum_{i=1}^{n}{\left(X_{i}-\bar{X}\right)}^{2}}}$$

(5) $$PBIAS=\frac{\sum_{i=1}^{n}\left(X_{i}-{\hat{X}}_{i}\right)\times 100}{\sum_{i=1}^{n}X_{i}}$$

where X_i_, ${\hat{X}}_{i}$, $\bar{X}$, and n represents the actual monitoring value, the modeled value, the average value of the actual monitoring value, and the number of monitoring samples, respectively.

Developed by the United States Environmental Protection Agency, human health risk assessment was used to calculate a non-carcinogenic hazards index for adult exposure to potentially toxic elements.

ADD_ing_ represents the average daily intake via ingestion:

(6) $$ADD_{ing}(mg\cdot kg^{-1}\cdot day^{-1})=C\times \frac{IngR\times EF\times ED}{BW\times AT}\times 10^{-6}$$

where C represents the potentially toxic element content (mg kg^−1^), and the maximum value of the potentially toxic elements was used to calculate the non-carcinogenic risk. IngR represents the ingestion rate (200 mg day^−1^) [55], EF represents the exposure frequency (350 day year^−1^) [56], ED represents the exposure duration (30 years) [56], BW represents the body weight (70 kg) [56], and AT represents the exposure time (AT = 365 × ED).

ADD_inh_ represents the average daily intake via inhalation:

(7) $$ADD_{inh}(mg\cdot kg^{-1}\cdot day^{-1})=C\times \frac{InhR\times EF\times ED}{PEF\times BW\times AT}$$

where InhR represents the inhalation rate (12.8 m^3^ day^−1^) [57] and PEF is the particle emission factor (1.36 × 10^9^ m^3^ kg^−1^) [55].

ADD_derm_ represents the average daily intake via dermal absorption:

(8) $$ADD_{derm}(mg\cdot kg^{-1}\cdot day^{-1})=C\times \frac{SA\times SL\times ABS\times EF\times ED}{BW\times AT}\times 10^{-6}$$

where SA represents the exposed skin area (4350 cm^2^) [57], SL is the skin adherence factor (0.2 mg cm^−2^ day^−1^) [55], and ABS represents the dermal absorption factor (dimensionless, ABS = 0.001). For As, ABS = 0.03 [55].

For exposure pathway i, non-carcinogenic hazards, such as a hazard quotient (HQ), are calculated with the rate of the corresponding reference dose for exposure pathway i (RfD_i_):

(9) $$HQ_{i}=ADD_{i}/RfD_{i}$$

The hazard index (HI) is calculated as follows:

(10) $$HI=\sum_{i=1}^{3}HQ_{i}$$

If HI < 1 or HQ < 1, it is suggested that there are no non-carcinogenic risks. If HI > 1 or HQ > 1, it is inferred that non-carcinogenic effects occurred [58].

## 3. Results and Discussions

### 3.1. Basic Statistical Results for the Contents of Major Elements and Potentially Toxic Elements

The contents for TOM and soil elements, including iron (Fe), arsenic (As), cadmium (Cd), cobalt (Co), chromium (Cr), copper (Cu), nickel (Ni), lead (Pb), antimony (Sb), thallium (Tl), vanadium (V), and zinc (Zn), and the health risk assessment for potentially toxic elements are shown in Table 1. The average content of Fe is 27.07 g kg^−1^, and the average value of TOM is 14.59 g kg^−1^ (Table 1). Among the potentially toxic elements, Hg, Cd, Sb, and Tl have the lowest average contents. In the Bosten Lake basin, the concentrations of TOM and soil elements are normally distributed (*p* > 0.05).

### 3.2. Influencing Factors for the Variation of Potentially Toxic Elements

**Figure 2:**
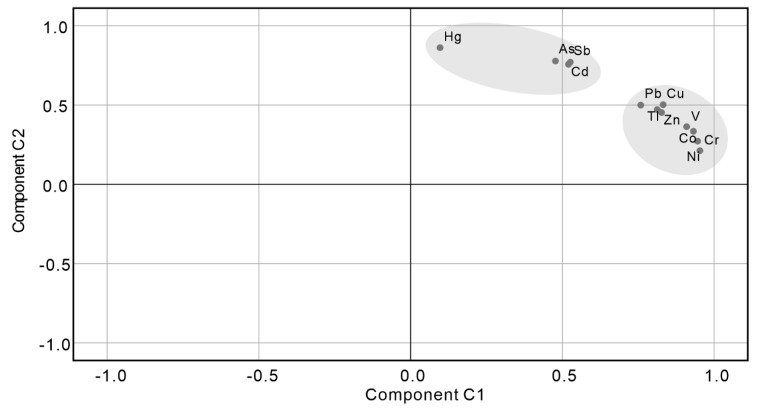
The component-loading plot of the potentially toxic elements, which indicates the potential influencing factors.

A principal component analysis was used to analyze the potential influencing factors for the variation of potentially toxic elements. Two components were extracted, which accounted for 89.2% of the total variance in the data set for potentially toxic elements (Table S1, supplementary electronic files). The potentially toxic elements were grouped according to their loadings (Figure 2). The first component accounted for 57.5% and formed a group composed of V, Ni, Cr, Co, Zn, Cu, Pb, and Tl, which had high loadings of V (0.91), Ni (0.95), Cr (0.95), Co (0.93), Zn (0.83), Cu (0.83), Pb (0.76), and Tl (0.81). The second component accounted for 31.7% and formed another group composed of Hg, Sb, Cd, and As, which had high loadings of Hg (0.86), Sb (0.77), Cd (0.76), and As (0.78) (Figure 2). Through principal component analysis, it is concluded that there are obvious differences in the influencing factors between the two groups of potentially toxic elements.

**Figure 3:**
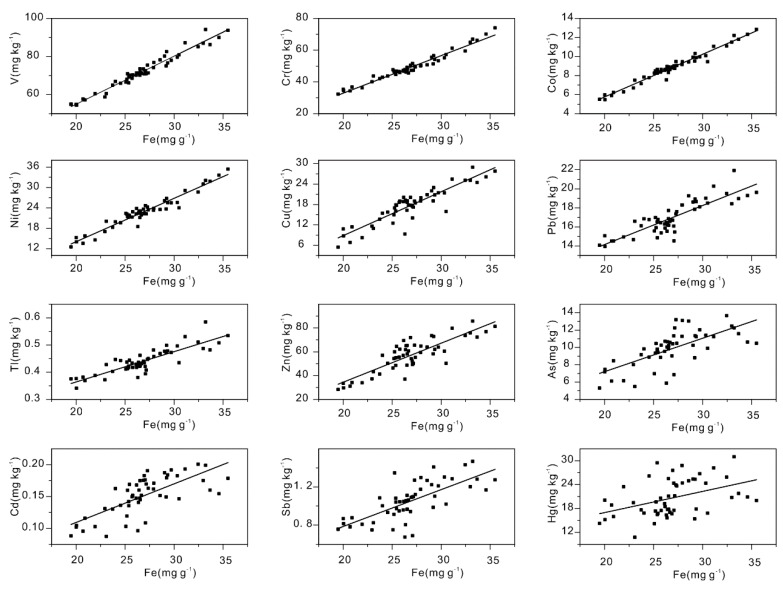
Scatter plots showing the potentially toxic elements (V, Cr, Co, Ni, Cu, Pb, Tl, Zn, As, Cd, Hg, and Sb) versus the content of Fe via a linear regression.

Fe is always used as a reference element for parent materials to calculate the enrichment of potentially toxic elements [59,60]. At a small scale, the background concentration gradients of the elements can be assumed to be uniform [61]. Coefficient of determinations for linear fitting were calculated among Fe and the potentially toxic elements (V: *r*^2^ = 0.95; Cr: *r*^2^ = 0.96; Co: *r*^2^ = 0.96; Ni: *r*^2^ = 0.93; Cu: *r*^2^ = 0.81; Pb: *r*^2^ = 0.73; Tl: *r*^2^ = 0.73; Zn: *r*^2^ = 0.70; As: *r*^2^ = 0.46; Cd: *r*^2^ = 0.47; Sb: *r*^2^ = 0.49; and Hg: *r*^2^ = 0.17) (Figure 3). The Pearson correlation coefficients with two-tailed significance tests were also calculated to reveal the inter-relationships among the Fe and the potentially toxic elements (V: *r* = 0.979, *p* < 0.001; Cr: *r* = 0.979, *p* < 0.001; Co: *r* = 0.982, *p* < 0.001; Ni: *r* = 0.965, *p* < 0.001; Cu: *r* = 0.903, *p* < 0.001; Pb: *r* = 0.857, *p* < 0.001; Tl: *r* = 0.867, *p* < 0.001; Zn: *r* = 0.850, *p* < 0.001; As: *r* = 0.692, *p* < 0.001; Cd: *r* = 0.719, *p* < 0.001; Sb: *r* = 0.710, *p* < 0.001; and Hg: *r* = 0.425, *p* = 0.0017). Notably, the contents of potentially toxic elements (Hg, Sb, Cd, and As) showed a relatively weaker correlation with Fe, and many samples showed a deviation during the simple linear regression, indicating that some potential influencing factor (e.g., TOM) other than the soil parent materials, had some impact on these four potentially toxic elements.

**Figure 4:**
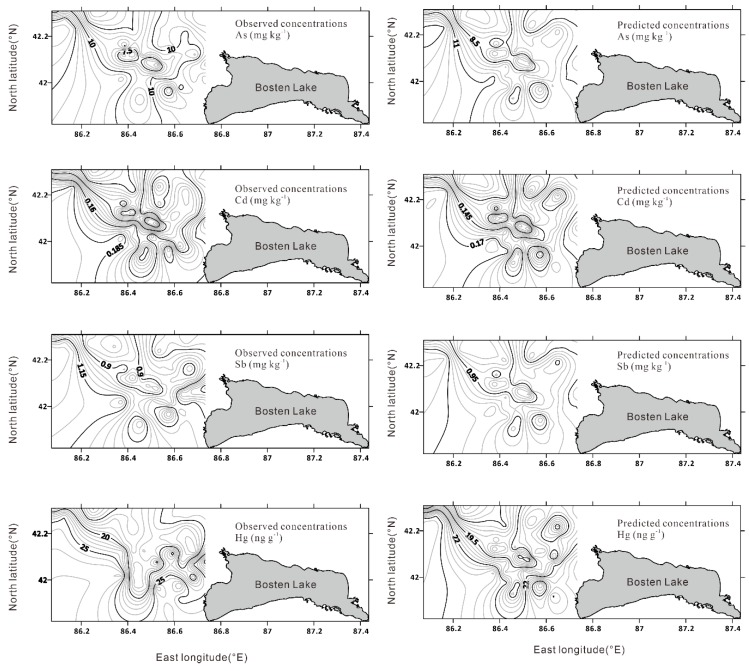
Geographically weighted regression predicted concentrations of potentially toxic elements compared with the observed/measured values in the Bosten Lake region.

To quantitatively analyse the relationships among the potentially toxic elements, Fe, and TOM in the soils, the potentially toxic elements (Hg, Sb, Cd, and As) were assigned as the dependent variable, and Fe and TOM were chosen as the independent variables in the models of the geographically weighted regression and the classical linear model. When modelling with the classical linear model (Figure 4), the fitting coefficients for the predicted values and actual measured values were found (Hg: *r*^2^ = 0.31; Sb: *r*^2^ = 0.64; Cd: *r*^2^ = 0.81; and As: *r*^2^ = 0.68). Due to the results of the geographically weighted regression, the correlation coefficients significantly improved (Hg: *r*^2^ = 0.56; Sb: *r*^2^ = 0.74; Cd: *r*^2^ = 0.89; and As: *r*^2^ = 0.85). Combined with the evaluation of the modeling results, which was based on the evaluation criterion, the results via the geographically weighted regression were acceptable (Table 2). The residuals from the results of the geographically weighted regression and classical linear model passed the normality test (Figure S1, supplementary electronic files). From a geographic perspective, the relationships among the potentially toxic elements, Fe and TOM had geographical or spatial heterogeneity, and the uniform values generated by the classical statistics ignored the geographical control on the distributions of potentially toxic elements.

**Figure 5:**
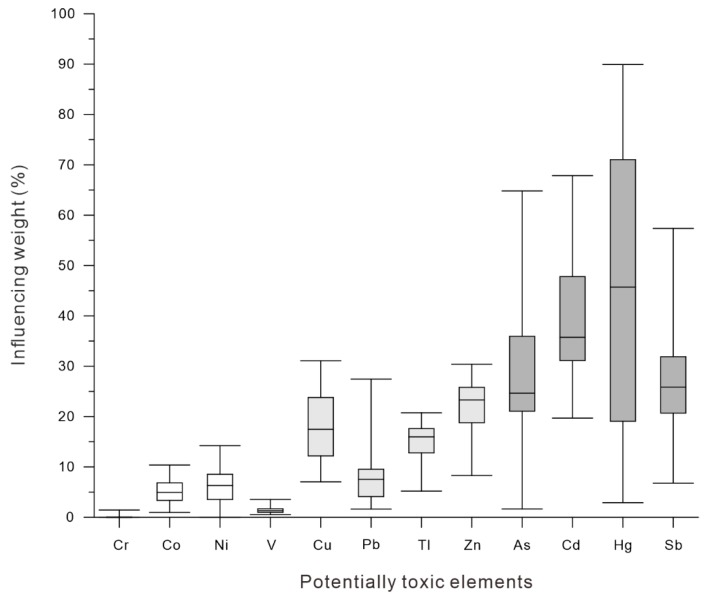
Statistical plots indicating the percentage of the potentially toxic elements (Cr, Co, Ni, V, Cu, Pb, Tl, Zn, As, Cd, Hg, and Sb) affected by soil organic matter, which show the minimum, maximum, median, lower quartile, and upper quartile values.

Based on the acceptance and validity of the geographically weighted regression, all of the potentially toxic elements As, Cd, Co, Cr, Cu, Ni, Pb, Sb, Tl, V, and Zn were simulated. By calculating the ratio of the part of the potentially toxic elements affected by the organic matter content, we can see that potentially toxic elements such as As, Cd, Hg, and Sb have average values of 28.8%, 39.2%, 46.5%, and 26.6% for the contents influenced by organic matter, respectively (Figure 5). The contents of potentially toxic elements are relatively low in soils, and changes in environmental conditions (e.g., climate, type, duration, and intensity of human activity) can easily have a profound impact on the distribution and content of elements. The main irrigation method in the oasis of this basin is drip irrigation. Existing studies have shown that fertilizers contain potentially toxic elements, such as Zn, Cu, Pb, Cd, As, and Hg [62,63,64], and a large amount of water-soluble fertilizer enters the soil via drip irrigation. Notably, the source of the influence of the organic matter has not yet been identified in this article. The potentially toxic elements affected by organic matter may come from human activities or from the process of soil formation under natural conditions.

Simply comparing the difference in heavy metal content between the study region and other regions has no practical significance. By calculating the health risks associated with potentially toxic elements, the extent of contamination in different regions can be reflected to some extent. Based on the risk assessment calculation for heavy metal pollution, the hazard quotient (HQ) via the ingestion (HQ_ing_) of surface soils are higher than those via inhalation (HQ_inh_) and dermal absorption (HQ_dermal_) (Table 3). Different from the degree of pollution in other economically developed regions around the world [65], the health risk index value for potentially toxic elements is less than one (Table 3), which is similar to that for the Issyk-Kul basin [66] and a suburban region of Bishkek [28] in the same arid region of Central Asia. Although this result reflects that the concentration of potentially toxic elements in arid areas has not reached a hazardous level, some potentially toxic elements (As, Cd, Sb, and Hg) have been significantly affected by the surface environment and they need to be paid enough attention.

## 4. Conclusions

Due to the lack of research on potentially toxic elements in soils in the arid region of Central Asia, a comprehensive study was conducted by analyzing Bosten Lake basin soils, and the method of geographically weighted regression provided a quantitative way to reveal possible influencing factors and model the distribution of potentially toxic elements. The detailed conclusions are as follows:

(1) Based on the calculations from the human health risk assessment, HI < 1 suggests that no significant non-carcinogenic risks to human health occurred in this region.

(2) At the basin scale, most potentially toxic elements had significant gradient changes. Potentially toxic elements (As, Cd, Hg, and Sb) were more susceptible to the organic matter in soils.

(3) Compared with the classical linear model, the modeling results were significantly improved with the geographically weighted regression, which suggested that spatial heterogeneity existed in the relationships among the potentially toxic elements and environmental variables.

(4) By calculating the ratio of the part of the potentially toxic elements affected by the organic matter content and that affected by the parent materials, the rates affected by organic matter were 28.8%, 39.2%, 46.5%, and 26.6% for the potentially toxic elements of As, Cd, Hg, and Sb, respectively.

## Figures and Tables

**Table 1:** Descriptive statistical analysis of major elements and potentially toxic elements in the surface soils of the Bosten Lake basin.

Composition	Unit	LOD ^b^	Basic Statistics				
			Minimum	Maximum	Average	Standard Deviation	Standard Error
TOM ^a^	g kg^−1^	0.01	4.46	23.43	14.59	5.17	0.75
Fe	g kg^−1^	0.005	19.50	35.50	27.07	3.50	0.51
V	mg kg^−1^	2	54.50	94.18	72.83	9.09	1.31
Zn	mg kg^−1^	0.1	28.30	85.80	58.00	13.71	1.98
Cr	mg kg^−1^	0.1	32.27	74.05	49.60	8.69	1.25
Co	mg kg^−1^	0.01	5.46	12.84	8.92	1.62	0.23
Ni	mg kg^−1^	0.05	12.53	35.40	23.09	4.75	0.69
Cu	mg kg^−1^	0.02	5.46	28.94	17.94	5.20	0.75
As	mg kg^−1^	0.1	5.30	13.67	9.86	2.02	0.29
Cd	mg kg^−1^	0.01	0.09	0.20	0.15	0.03	0.00
Sb	mg kg^−1^	0.05	0.68	1.47	1.05	0.20	0.03
Tl	mg kg^−1^	0.02	0.34	0.58	0.44	0.05	0.01
Pb	mg kg^−1^	0.01	13.95	21.93	17.05	1.71	0.25
Hg	ng g^−1^	0.01	10.75	31.00	20.62	4.67	0.67

^a^: The content of total organic matter; ^b^: Limit of detection.

**Table 2:** General performance ratings for the results of the geographically weighted regression and multiple classical linear models for potentially toxic elements (PTEs).

PTEs	Model Equation	RSE ^c^	NSE ^d^	PBIAS ^e^	Performance Rating ^f^
As ^a^	[As] = 2.04 × 10^−1^ × [Fe] + 2.23 × 10^−1^ × [TOM] + 1.10	0.57	0.68	0.00	Good
Cd ^a^	[Cd] = 2.42 × 10^−3^ × [Fe] + 4.15 × 10^−3^×[TOM] + 2.56 × 10^−2^	0.44	0.81	−0.10	Very good
Sb ^a^	[Sb] = 2.39 × 10^−2^ × [Fe] + 1.85 × 10^−2^ × [TOM] + 1.36 × 10^−1^	0.60	0.64	−0.001	Satisfactory
Hg ^a^	[Hg] = 1.85 × 10^−1^ × [Fe] + 4.23 × 10^−1^ × [TOM] + 9.45	0.83	0.31	0.00	Unsatisfactory
As ^b^	[As]^e^ = [β_1_]_As_ × [Fe] + [β_2_]_As_ × [TOM] + [β_0_]_As_	0.39	0.85	0.03	Very good
Cd ^b^	[Cd]^e^ = [β_1_]_Cd_ × [Fe] + [β_2_]_Cd_ × [TOM] + [β_0_]_Cd_	0.34	0.88	0.13	Very good
Sb ^b^	[Sb]^e^ = [β_1_]_Sb_ × [Fe] + [β_2_]_Sb_ × [TOM] + [β_0_]_Sb_	0.51	0.74	−0.20	Good
Hg ^b^	[Hg]^e^ = [β_1_]_Hg_ × [Fe] + [β_2_]_Hg_ × [TOM] + [β_0_]_Hg_	0.68	0.54	−0.36	Satisfactory

^a^: Classical linear model; ^b^: Geographically weighted regression, the detailed parameters are shown in Table S2 (supplementary electronic files); ^c^: Root mean square error; ^d^: Nash–Sutcliffe efficiency; ^e^: Percentage bias; ^f^: Performance ratings followed common criteria of the reference [54].

**Table 3:** Human health risk assessment for potentially toxic elements (PTEs) in the surface soils of the Bosten Lake basin.

PTEs	Maximum	Reference Dose for Exposure Pathway (RfD_i_) [55] (mg kg^−1^ day^−1^)			Non-Carcinogenic Hazards Index			
		RfD_ing_	RfD_dermal_	RfD_inh_	HQ_ing_	HQ_inh_	HQ_dermal_	HI
V	13.7 mg kg^−1^	7.00 × 10^−3^	7.00 × 10^−5^	7.00 × 10^−3^	3.69 × 10^−2^	1.73 × 10^−6^	1.60 × 10^−2^	5.29 × 10^−2^
Zn	0.2 mg kg^−1^	3.00 × 10^−1^	6.00 × 10^−^^2^	3.00 × 10^−1^	0	3.69 × 10^−8^	1.70 × 10^−5^	8.01 × 10^−4^
Cr	1.5 mg kg^−1^	3.00 × 10^−3^	6.00 × 10^−^^5^	2.86 × 10^−5^	6.76 × 10^−2^	3.34 × 10^−4^	1.47 × 10^−2^	8.27 × 10^−2^
Co	0.6 mg kg^−1^	2.00 × 10^−2^	1.60 × 10^−2^	5.69 × 10^−^^6^	1.76 × 10^−3^	2.91 × 10^−4^	9.56 × 10^−6^	2.06 × 10^−3^
Ni	21.9 mg kg^−1^	2.00 × 10^−2^	5.40 × 10^−3^	2.00 × 10^−2^	4.85 × 10^−3^	2.28 × 10^−7^	7.81 × 10^−5^	4.93 × 10^−3^
Cu	31.0 mg kg^−1^	4.00 × 10^−2^	1.20 × 10^−2^	4.00 × 10^−2^	1.98 × 10^−3^	9.33 × 10^−8^	2.87 × 10^−5^	2.01 × 10^−3^
As	23.4 mg kg^−1^	3.00 × 10^−4^	1.23 × 10^−^^4^	3.00 × 10^−4^	1.25 × 10^−1^	5.88 × 10^−6^	3.97 × 10^−2^	1.65 × 10^−1^
Cd	35.5 mg kg^−1^	1.00 × 10^−3^	1.00 × 10^−^^5^	1.00 × 10^−3^	5.50 × 10^−4^	2.59 × 10^−8^	2.39 × 10^−4^	7.89 × 10^−4^
Sb	94.2 mg kg^−1^	4.00 × 10^−4^	8.00 × 10^−6^	4.01 × 10^−4^	1.01 × 10^−2^	4.73 × 10^−7^	2.19 × 10^−3^	1.22 × 10^−2^
Tl	85.8 mg kg^−1^	8.00 × 10^−^^5^	1.00 × 10^−5^	8.00 × 10^−5^	2.00 × 10^−2^	9.42 × 10^−7^	6.96 × 10^−4^	2.07 × 10^−2^
Pb	74.1 mg kg^−1^	3.50 × 10^−3^	5.25 × 10^−4^	3.51 × 10^−3^	1.72 × 10^−2^	8.06 × 10^−7^	4.98 × 10^−4^	1.77 × 10^−2^
Hg	12.8 ng g^−1^	3.00 × 10^−4^	2.10 × 10^−5^	3.00 × 10^−4^	2.83 × 10^−4^	1.33 × 10^−8^	1.76 × 10^−5^	3.01 × 10^−4^

## Supplementary Materials

The following are available online at https://www.mdpi.com/1660-4601/16/19/3741/s1, Table S1: Total variance explained for potentially toxic elements with Principal Component Analysis; Table S2: The detailed parameters for geographically weighted regression in Table 2; Figure S1: Residuals normality test of the residuals for the fitting results of potentially toxic elements (As, Cd, Sb, and Hg) with geographically weighted regression and classical linear model.
