# Supplementary material for: Spatial Distribution and Health Risk Assessment of Potentially Toxic Elements in Surface Soils of Bosten Lake Basin, Central Asia
Source: Int J Environ Res Public Health. 2019 Oct 4;16(19):3741. doi: 10.3390/ijerph16193741 (PMC6801520; doi:10.3390/ijerph16193741)
Supplement: Supplementary file 1 [file ijerph-16-03741-s001.pdf]

Supplementary information

Table S1. Total variance explained for potentially toxic elements with Principal Component Analysis.

Component	Initial Eigenvalues			Extraction Sums of Squared Loadings		
	Total	% of Variance	Cumulative %	Total	% of Variance	Cumulative %
1	9.6	79.6	79.6	6.9	57.5	57.5
2	1.1	9.6	89.2	3.8	31.7	89.2
3	0.6	4.7	93.8			
4	0.3	2.1	95.9			
5	0.2	1.6	97.6			
6	0.1	0.9	98.5			
7	0.1	0.7	99.2			
8	0.0	0.4	99.6			
9	0.0	0.2	99.8			
10	0.0	0.2	99.9			
11	0.0	0.0	100.0			
12	0.0	0.0	100.0			

Table S2. The detailed parameters for geographically weighted regression in Table 2.

Sample NO	$[\beta_1]_{As}$	$[\beta_2]_{As}$	$[\beta_0]_{As}$	$[\beta_1]_{Cd}$	$[\beta_2]_{Cd}$	$[\beta_0]_{Cd}$	$[\beta_1]_{Sb}$	$[\beta_2]_{Sb}$	$[\beta_0]_{Sb}$	$[\beta_1]_{Hg}$	$[\beta_2]_{Hg}$	$[\beta_0]_{Hg}$
1	8.94E-01	2.95E-01	-1.73E+01	5.96E-03	4.76E-03	-8.54E-02	5.61E-02	2.54E-02	-7.78E-01	2.05E+00	3.39E-01	-3.92E+01
2	8.81E-01	3.00E-01	-1.70E+01	6.20E-03	4.63E-03	-8.99E-02	5.37E-02	2.60E-02	-7.20E-01	2.05E+00	3.41E-01	-3.90E+01
3	7.41E-01	3.31E-01	-1.38E+01	5.61E-03	4.56E-03	-7.19E-02	3.43E-02	2.98E-02	-2.60E-01	1.72E+00	3.70E-01	-3.07E+01
4	5.07E-01	3.66E-01	-8.11E+00	4.16E-03	4.64E-03	-3.33E-02	2.12E-02	3.15E-02	5.38E-02	1.09E+00	4.03E-01	-1.42E+01
5	2.98E-01	3.87E-01	-3.03E+00	3.12E-03	4.64E-03	-5.16E-03	9.87E-03	3.20E-02	3.30E-01	6.91E-01	4.01E-01	-3.87E+00
6	2.66E-01	3.92E-01	-2.27E+00	3.00E-03	4.59E-03	-1.93E-03	7.41E-03	3.25E-02	3.86E-01	6.53E-01	4.15E-01	-3.21E+00
7	3.08E-02	3.25E-01	3.98E+00	1.03E-03	4.72E-03	4.91E-02	6.84E-03	2.57E-02	4.52E-01	2.02E-01	2.25E-01	1.03E+01
8	1.79E-01	2.35E-01	1.20E+00	2.44E-03	4.43E-03	2.20E-02	1.98E-02	1.79E-02	2.24E-01	6.85E-01	3.44E-02	2.18E+00
9	9.82E-02	2.63E-01	2.87E+00	1.51E-03	4.63E-03	4.10E-02	1.33E-02	2.05E-02	3.49E-01	3.16E-01	1.44E-01	9.18E+00
10	8.55E-02	2.65E-01	3.20E+00	1.30E-03	4.61E-03	4.61E-02	1.28E-02	2.06E-02	3.62E-01	2.40E-01	1.61E-01	1.06E+01
11	2.01E-01	2.23E-01	8.82E-01	2.55E-03	4.24E-03	2.18E-02	2.12E-02	1.73E-02	2.06E-01	4.90E-01	1.45E-01	5.72E+00
12	1.27E-01	2.39E-01	2.54E+00	1.64E-03	4.37E-03	4.15E-02	1.65E-02	1.87E-02	2.99E-01	2.24E-01	1.92E-01	1.10E+01
13	9.40E-02	2.42E-01	3.33E+00	1.22E-03	4.30E-03	5.15E-02	1.47E-02	1.89E-02	3.38E-01	1.57E-01	1.64E-01	1.24E+01
14	1.64E-01	2.31E-01	1.72E+00	2.11E-03	4.31E-03	3.14E-02	1.88E-02	1.81E-02	2.53E-01	3.31E-01	1.87E-01	8.86E+00
15	2.53E-01	2.05E-01	-1.50E-01	3.05E-03	4.08E-03	1.20E-02	2.52E-02	1.57E-02	1.29E-01	6.34E-01	1.19E-01	2.75E+00
16	3.12E-01	1.77E-01	-1.22E+00	3.58E-03	3.78E-03	3.66E-03	2.99E-02	1.38E-02	4.18E-02	6.99E-01	1.37E-01	1.23E+00
17	1.57E-01	2.16E-01	2.16E+00	1.87E-03	4.13E-03	3.93E-02	1.93E-02	1.73E-02	2.54E-01	1.73E-01	2.38E-01	1.19E+01
18	2.78E-01	1.91E-01	-5.44E-01	3.41E-03	3.82E-03	6.68E-03	2.70E-02	1.52E-02	9.84E-02	4.52E-01	2.49E-01	5.69E+00
19	1.82E-01	2.08E-01	1.65E+00	2.20E-03	4.04E-03	3.29E-02	2.13E-02	1.66E-02	2.18E-01	1.87E-01	2.75E-01	1.13E+01
20	2.33E-01	1.97E-01	5.48E-01	2.92E-03	3.87E-03	1.78E-02	2.46E-02	1.58E-02	1.50E-01	2.50E-01	3.25E-01	9.46E+00
21	2.11E-01	1.91E-01	1.21E+00	2.55E-03	3.84E-03	2.72E-02	2.36E-02	1.56E-02	1.79E-01	1.53E-01	3.48E-01	1.14E+01
22	2.95E-01	1.79E-01	-7.53E-01	3.74E-03	3.59E-03	1.64E-03	2.83E-02	1.49E-02	7.37E-02	3.18E-01	3.62E-01	7.62E+00
23	1.70E-01	1.59E-01	2.71E+00	1.85E-03	3.42E-03	4.85E-02	2.16E-02	1.37E-02	2.45E-01	1.27E-01	2.04E-01	1.29E+01
24	1.88E-01	1.77E-01	2.02E+00	2.12E-03	3.69E-03	3.92E-02	2.23E-02	1.50E-02	2.18E-01	1.09E-01	2.99E-01	1.27E+01
25	2.10E-01	1.83E-01	1.36E+00	2.50E-03	3.75E-03	2.94E-02	2.38E-02	1.53E-02	1.82E-01	1.23E-01	3.62E-01	1.19E+01
26	1.88E-01	1.64E-01	2.21E+00	2.09E-03	3.53E-03	4.18E-02	2.26E-02	1.42E-02	2.22E-01	1.04E-01	2.88E-01	1.28E+01
27	2.99E-01	1.71E-01	-7.02E-01	3.97E-03	3.40E-03	-1.54E-03	2.89E-02	1.50E-02	6.36E-02	2.04E-01	4.65E-01	9.13E+00
28	3.29E-01	1.62E-01	-1.34E+00	4.34E-03	3.25E-03	-8.27E-03	3.05E-02	1.47E-02	2.66E-02	2.33E-01	4.74E-01	8.49E+00
29	3.74E-01	1.28E-01	-1.92E+00	4.76E-03	2.80E-03	-1.13E-02	3.26E-02	1.36E-02	-8.02E-03	2.02E-01	5.02E-01	9.37E+00
30	2.61E-01	1.75E-01	2.21E-01	3.38E-03	3.54E-03	1.11E-02	2.70E-02	1.49E-02	1.10E-01	1.40E-01	4.63E-01	1.05E+01
31	2.10E-01	1.50E-01	1.92E+00	2.39E-03	3.40E-03	3.68E-02	2.42E-02	1.36E-02	1.97E-01	8.51E-02	3.73E-01	1.24E+01
32	1.96E-01	1.05E-01	2.94E+00	2.10E-03	2.87E-03	5.05E-02	2.43E-02	1.04E-02	2.28E-01	1.44E-01	2.31E-01	1.20E+01
33	2.23E-01	1.60E-01	1.45E+00	2.64E-03	3.50E-03	2.98E-02	2.49E-02	1.42E-02	1.73E-01	8.18E-02	4.22E-01	1.21E+01
34	2.07E-01	1.41E-01	2.13E+00	2.33E-03	3.30E-03	3.97E-02	2.41E-02	1.31E-02	2.05E-01	8.99E-02	3.52E-01	1.24E+01
35	2.99E-01	1.63E-01	-5.46E-01	4.14E-03	3.24E-03	-3.66E-03	2.91E-02	1.52E-02	5.88E-02	1.18E-01	5.58E-01	1.01E+01
36	3.36E-01	1.52E-01	-1.31E+00	4.73E-03	2.93E-03	-1.34E-02	3.14E-02	1.51E-02	4.77E-03	8.81E-02	6.06E-01	1.06E+01
37	2.54E-01	1.57E-01	7.16E-01	3.26E-03	3.38E-03	1.62E-02	2.68E-02	1.46E-02	1.24E-01	7.06E-02	5.30E-01	1.12E+01
38	2.41E-01	1.50E-01	1.17E+00	2.98E-03	3.35E-03	2.35E-02	2.62E-02	1.41E-02	1.48E-01	6.37E-02	5.05E-01	1.16E+01
39	2.17E-01	8.65E-02	2.75E+00	2.40E-03	2.76E-03	4.57E-02	2.56E-02	1.02E-02	2.11E-01	1.16E-01	3.69E-01	1.12E+01

40	2.10E-01	3.43E-02	3.72E+00	2.22E-03	2.21E-03	5.81E-02	2.70E-02	5.78E-03	2.29E-01	1.97E-01	2.54E-01	1.01E+01
41	2.27E-01	1.07E-01	2.21E+00	2.67E-03	2.97E-03	3.67E-02	2.58E-02	1.20E-02	1.88E-01	8.09E-02	4.70E-01	1.11E+01
42	3.05E-01	1.55E-01	-5.51E-01	4.57E-03	3.00E-03	-1.12E-02	2.98E-02	1.57E-02	3.82E-02	4.88E-02	6.67E-01	1.04E+01
43	2.84E-01	1.51E-01	8.21E-02	4.09E-03	3.12E-03	-7.59E-04	2.86E-02	1.55E-02	7.29E-02	4.80E-02	6.55E-01	1.04E+01
44	2.57E-01	1.39E-01	9.62E-01	3.42E-03	3.17E-03	1.54E-02	2.71E-02	1.46E-02	1.22E-01	4.98E-02	6.05E-01	1.07E+01
45	2.40E-01	1.19E-01	1.72E+00	3.02E-03	3.05E-03	2.71E-02	2.64E-02	1.34E-02	1.59E-01	6.35E-02	5.59E-01	1.07E+01
46	2.09E-01	7.52E-03	4.26E+00	2.19E-03	2.00E-03	6.35E-02	2.78E-02	4.32E-03	2.36E-01	2.34E-01	3.06E-01	8.40E+00
47	2.78E-01	1.41E-01	3.93E-01	4.27E-03	3.02E-03	-4.38E-03	2.88E-02	1.61E-02	6.34E-02	5.19E-02	7.25E-01	9.25E+00
48	2.56E-01	1.19E-01	1.33E+00	3.59E-03	3.01E-03	1.34E-02	2.75E-02	1.48E-02	1.15E-01	7.80E-02	6.68E-01	9.00E+00

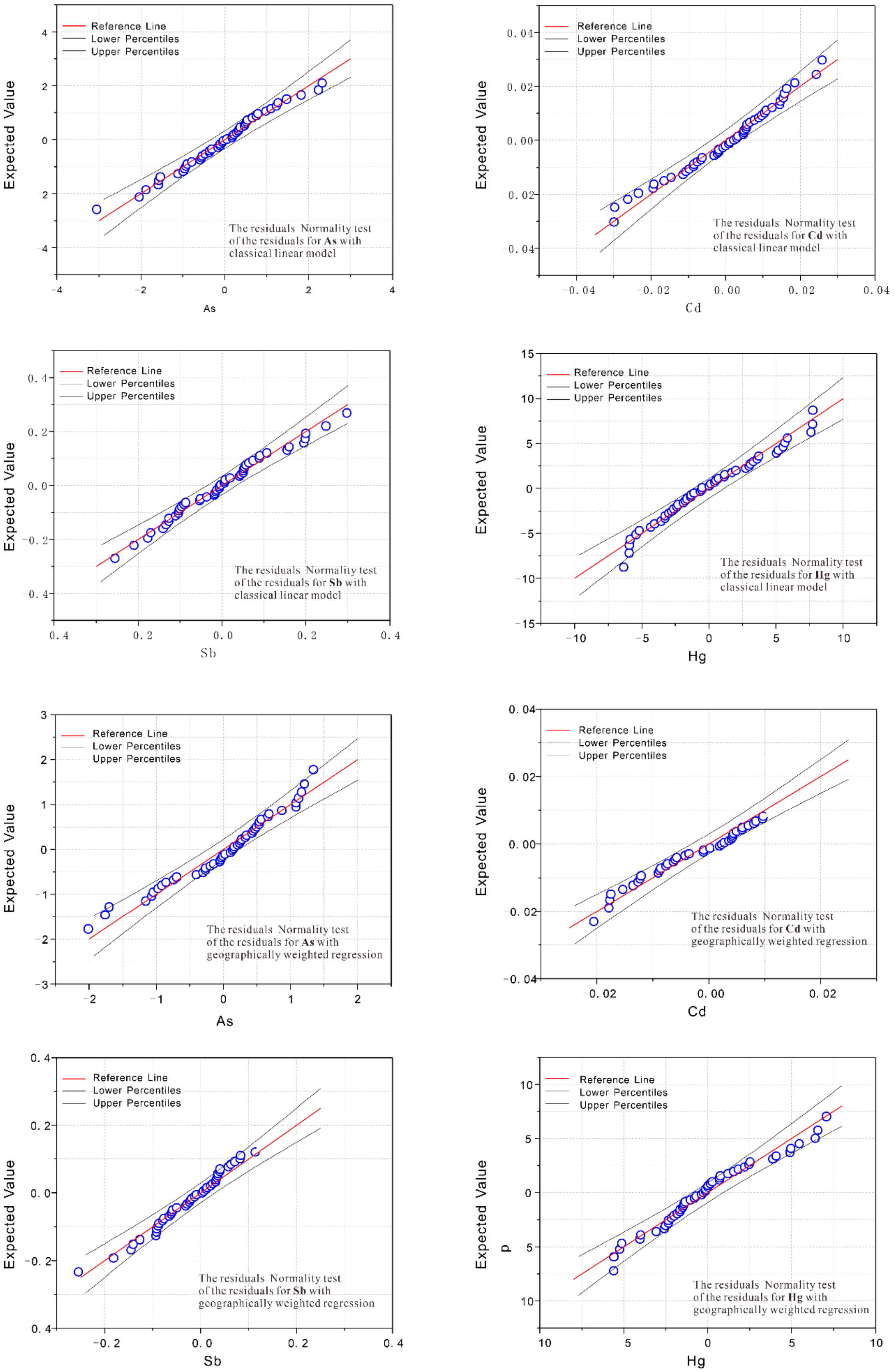

Figure S1. Residuals normality test of the residuals for the fitting results of potentially toxic elements (As, Cd, Sb and Hg) with geographically weighted regression and classical linear model.
